# Anti-inflammatory treatment using alpha melanocyte stimulating hormone (α-MSH) does not alter osteoblasts differentiation and fracture healing

**DOI:** 10.1186/s12891-025-08374-9

**Published:** 2025-02-06

**Authors:** Johanna Graue, Melanie Timmen, Katharina Schmitz, Daniel Kronenberg, Markus Böhm, Kishor K. Sivaraj, M. Gabriele Bixel, Richard Stange

**Affiliations:** 1 Department of Regenerative Musculoskeletal Medicine, Institute of Musculoskeletal Medicine, University of Muenster, Albert-Schweitzer-Campus 1, D3, 48149 Muenster, Germany; 2 Department of Dermatology, University Hospital Muenster, Von-Esmarch-Str. 58, 48149 Muenster, Germany; 3 Max Planck Institute for Molecular Biomedicine, Röntgenstraße 20, 48149 Muenster, Germany

**Keywords:** Melanocortin receptor, Inflammation, Fracture healing, Osteoblast, Alpha-MSH

## Abstract

**Background:**

Alpha-melanocyte-stimulating-hormone (α-MSH) has been identified as a new anti-inflammatory treatment compound in rheumatoid arthritis (RA) and other inflammatory diseases. However, its direct effect on bone cell differentiation or on bone regeneration, which is an inflammatory process, too, has not been investigated, yet. Bone tissue is significantly affected in inflammatory joint diseases. Additionally, inflammatory signaling is essential -in bone regeneration during fracture healing. Therefore, we evaluated the impact of α-MSH-treatment on bone forming cells in an inflammatory setting in vitro and as a treatment approach in a murine fracture healing model in vivo.

**Methods:**

The influence of α-MSH treatment and melanocortin-receptor expression patterns was investigated in vitro in the presence of either IL-1β or/and TNF-α as an inflammatory stimulus. Osteoblast cell function was evaluated by analyzing proliferation and mineralisation capacities. Using quantitative real time PCR, we analyzed mRNA expression of receptors. To explore the impact of α-MSH on bone regeneration in vivo, treatment with α-MSH or NaCl (control) was performed in a murine fracture-healing model using a closed femur fracture stabilized with an intramedullary implant (female, *n* = 6–8 mice per group).

**Results:**

α-MSH-treatment did not impair either proliferation nor mineralisation of osteoblastic cells under native or inflammatory conditions (no significant differences found). All four melanocortin receptor-molecules were expressed in murine osteoblastic cells but in very limited amounts and this did not change upon treatment with inflammatory cytokines or α-MSH or both at the same time. Callus formation in fractured femurs of α-MSH-treated mice was slightly delayed at day 14 post fracture with regard to less cartilage formation (NaCl: 19.9%; α-MSH: 13.5%) and soft tissue remodeling (NaCl: 15.2%; α-MSH: 19.5%) but these results were not significantly different and fracture healing overall occurred in a regular way.

**Conclusion:**

α-MSH has no negative impact on bone or bone-forming cells in native, inflammatory, or regenerative contexts. We can conclude from our results, that treatment of inflammatory diseases using α-MSH does not interfere significantly with bone regeneration in a murine fracture model and therefore treatment with α-MSH could be continued without negative effects on bone formation and bone regeneration in patients.

**Clinical trial number:**

Not applicable.

**Supplementary Information:**

The online version contains supplementary material available at 10.1186/s12891-025-08374-9.

## Background

Alpha-melanocyte-stimulating hormone (α-MSH) is a tridecapeptide derived from its precursor hormone proopiomelanocortin (POMC) and is the most important melanocyte-stimulating hormone (MSH), also known as melanotropin, that stimulates melanogenesis, a process primarily responsible for the pigmentation of hair and skin in mammals (including humans) [1, 2]. The melanocortin system includes the ligands α-, β-, and γ-MSH, as well as adrenocorticotropic hormone (ACTH), all of which share a central sequence His-Phe-Arg-Trp (HFRW) as a common pharmacophore. These ligands bind to their specific melanocortin receptor (MCR) molecules, five of which are known (MC1-5R) and differ in their affinity for ligands [3, 4]. These receptor molecules are G-protein-coupled receptors located in the cytoplasmic membrane with seven transmembrane domains. Furthermore, two endogenous antagonists, an agouti-signaling protein and an agouti-related peptide, are known.

In recent years, evidence has increased for alternative functions of α-MSH in addition to skin pigmentation, such as energy metabolism, food intake, sexual activity, exocrine secretion, collagen turnover in the skin, and inflammation [5, 6]. The anti-inflammatory effects of α-MSH in vitro include the inhibition of NFκB (nuclear factor ‘kappa-light-chain-enhancer’ of activated B cells), the suppression of proinflammatory cytokines such as tumor necrosis factor (TNF)-α, interleukin (IL)-1β, IL-6, and IL-8, the suppression of noncytokine proinflammatory mediators, and the modulation of regulatory T cells [5]. This phenomenon has been confirmed in animal models of inflammation, such as irritant and allergic contact dermatitis, cutaneous vasculitis, asthma, inflammatory bowel disease, and ocular and brain inflammation [7–10]. In the musculoskeletal context, an effect of α-MSH during rheumatoid arthritis and osteoarthritis has been described [11–14]. Böhm et al. further summarized evidence for the presence of MCRs in osteoarticular tissue, such as growth plate chondrocytes, human articular chondrocytes, and human osteoblasts [4]. However, studies examining the expression of MCR molecules or the impact of melanocortins on bone cells, such as osteoblasts and their functions, are very rare and often focused on cell lines, undifferentiated cells, or specialized investigations into a single receptor or ligand [15–17].

Rheumatoid arthritis (RA) is a disease characterized by joint pain and swelling, stiffness, systemic comorbidities, disability, reduced quality of life, and increased mortality caused by chronic, systemic inflammation. Moreover, this pathological process affects bone through an imbalance of increased osteoclast-mediated bone resorption and decreased osteoblast-driven bone formation [18, 19]. RA patients display resulting trabecular, periarticular, and systemic bone loss with focal bone erosions; a higher incidence of osteoporosis; and an increased fracture risk [20]. In addition, systemic chronic inflammation impairs the acute inflammatory process during early fracture healing and may favor delayed fracture healing, which is more frequent in patients with RA [21, 22]. Anti-inflammatory TNF blockers, such as infliximab, etanacerpt, and adalimumab, represent breakthroughs in the treatment of RA. However, due to the high variety of patients, some patients experience treatment failure, necessitating the development of new drugs. αMSH has been shown to be a promising candidate due to its anti-inflammatory properties as described before. In vivo, several arthritis models using MCR agonists like αMSH exhibited reduced disease activity and severity according to clinical scoring and paw swelling, as well as reduced synovitis via the MC1R and MC3R pathways, indicating the existence of an endogenous anti-inflammatory tissue-protective circuit involving the melanocortin system [23–26]. To date, it remains unclear which cell type produces melanotropins such as α-MSH in joints or bone tissue or which cell type actively reacts to inflammation via the MCR system. Studies on inflammation, α-MSH and cells of the musculoskeletal system have mostly focused on synovial fibroblasts and chondrocytes [13, 14, 27–30]. Recently, it was shown that osteoblastic activity was increased in the presence of α-MSH in the osteoblast-like cell line MC3T3E and in an osteoblast system in goldfish [17, 31]. However, to date there is missing evidence for the effect of α-MSH on bone and bone regeneration. Due to its anti-inflammatory properties, treatment with α-MSH may comprise a potential risk of adverse effects on fracture healing. This study, therefore, aimed to shed light on the role of α-MSH and its receptors in osteoblastic cell differentiation and regeneration in physiological and inflammatory environments and to reveal whether treatment with α-MSH affects bone cells and bone regeneration.

## Materials and methods

### Animals

Fracture healing was examined in 12-week-old female C57BL/6J mice (originated from Charles River, France). All experimental protocols were approved by the Landesamt für Naturschutz, Umweltschutz und Verbraucherschutz (LANUV, reference numbers: 84-02.04.2014.A434; 84-02.05.50.A15.005), North Rhine-Westphalia, Germany, and performed according to governmental guidelines for the use of experimental animals and as well as the ARRIVE guidelines. Mice were bred in the central animal experimental unit of the University Hospital Muenster (ZTE) and kept with a 12-hour light/dark cycle, up to 65% relative humidity and 20 °C temperature in cages with groups of up to 4 animals enriched with nesting material and paper houses. The mice had access to tap water and standard rodent chow (Altromin GmbH, Lage, Germany) ad libitum.

### Surgical procedure

Female 12-week-old mice (*n* = 10 each) were anesthetized with xylazine (2%, 12 mg/kg)/ketamine (10%, 75 mg/kg) intraperitoneally (i.p.). The skin and fascia of the left knee were incised over the patellar tendon, and the patellar tendon was split medially. The femoral medullary cavity was opened intercondylar using a 0.5 mm drill. After a guide wire was retrogradely inserted into the medullary canal of the left femur, a standardized mid-shaft fracture was generated using a 3-point bending device [32, 33], and an intramedullary MouseScrew (RIS System, Landquart, Switzerland) was implanted [34]. Skin and fascia were closed by single-button sutures. After the surgery and 14 days postoperatively, the fracture was verified via X-ray. Mice with dislodged or displaced fractures were excluded. Mice received Rimadyl (5 µg/kg body weight, Zoetis) as an analgesic. The mice were injected daily (i.p.) with either 100 µl of 0.9% NaCl or [Nle4, DPhe7]-(NDP)-α-MSH (Bachem, 0.01 mg/ml in 0.9% NaCl). NDP-alpha-MSH is a synthetic analog of alpha-MSH that was developed in 1980 [35] and is highly potent and shows prolonged biological activity [36–38]. The concentrations of α-MSH chosen here has already been proven to be effective in mice [39]. The animals were euthanized by cervical dislocation after 14 days. Both femora were isolated, the screw was removed from the fractured femur, and both femora were fixed for 24 h in 4% paraformaldehyde (PFA).

### X-ray

The skeletal structure was visualized using a small animal X-ray system (In-Vivo DXS PRO, Carestream Health, Rochester, NY, USA). Pictures were taken after the mice were sacrificed at 35 kV for 1 min.

### µCT analysis

Both femora were scanned in a µCT imaging system (Bruker SkyScan 1172, Kontich, Belgium) with a resolution of 9 μm. Subsequently, the datasets were reconstructed using the SkyScan Software NRecon (beam hardening correction: 6, ring artifact reduction: 4, smoothing: 1) and DataViewer. The bone structure of the callus was analysed using the software CTAnalyser (Bruker). Native bone: Threshold-based segmentation (grey scale: 70–255) was used to differentiate between bone marrow, cortical bone and trabecular bone as follows: cortical bone: 100 to 255; trabecular bone: 70 to 100. Fractured bones: Segmentation was used to differentiate between soft tissue, cortical bone/fragments and newly formed trabecular bone as follows: osseous structures were identified by an adaptive threshold of 40 to 125, and cortical structures were excluded using a morphological escalator (Bruker MicroCT method note 116: MicroCT analysis of bone fracture callus and healing). Parameters such as tissue volume, bone volume, BV/TV, trabecular number, trabecular thickness and trabecular separation were used to characterize the tissue structure of the native bone and callus. Mice with dislodged or displaced fractures were excluded. The results are shown as the mean and SD. Individual values appear as data points in each graph.

### Histomorphometric analysis

Left fractured femora were decalcified for eight weeks in 20% EDTA, pH 7.5. Subsequently, the bones were embedded in paraffin, cut into 5 μm thick sagittal slices and stained according to the standard protocol of Alcian blue staining. Mice with dislodged or displaced fractures were excluded. Digital image analysis was used to quantify the area of the total callus, trabecular callus, cartilaginous callus and soft tissue using a BX51 microscope and cell sense dimensions software (CellSens software, Olympus, Muenster, Germany).

### Cell isolation and culture

Osteoprogenitor cells were isolated from the calvaria of 3- to 5-day-old mice as described previously [40, 41]. Afterwards, cells were seeded at a density of 5 × 10^3^ cells per well in 96-well plates for MTT assays and 2.5 × 10^4^ cells per well in 24-well plates for RNA isolation and Alizarin Red staining and cultured overnight in α-MEM supplemented with 10% FCS + 100 U/ml penicillin and 2 mM L-glutamine. Differentiation toward osteoblasts was induced using differentiation medium (DM) containing 10 nM dexamethasone, 0.2 mM L-ascorbate-2-phosphate and 10 mM ß-glycerophosphate (day 0 of differentiation). The α-MSH treatment medium also contained 1 µM NDP-α-MSH (Bachem) as previously described [14]. This concentration was shown to be effective on different cell types as described by cell culture experiments or activity assays [13, 15, 46 To simulate inflammatory conditions, either IL-1β (Cat. No:211-11B, PeproTech, Hamburg, Germany), TNF-α (Cat. No:410-MT, Biotechne, Wiesbaden, Germany) or both were added (10 ng/ml). The media were changed every other day. The cells were cultured for up to 25 days.

### RNA isolation, cDNA synthesis and qPCR

RNA was isolated on days 1, 3, 7, 14 and 25 with RLT buffer according to the manufacturer’s instructions using the Qiagen RNeasy Kit and the Quick-Start protocol provided by Qiagen. Afterwards, cDNA was synthesized with the Applied Biosystems RNA-to-cDNA Kit using 500 ng of RNA. The sequences of primers used for qPCR are listed in Table 1, and qPCR was conducted with a DyNAmo Flash SYBR Green qPCR Kit (Biozym, Hessisch Oldendorf, Germany) in a Bio-Rad IQ5 thermocycler with the corresponding IQ2 detection module (Munich, Germany).

**Table 1:** Sequences of primers used for quantitative real-time PCR

Gene	Primer sequence
HPRT sense	tgatagatccattcctatgactgtaga
HPRT antisense	aagacattctttccagttaaagttgag
MC1R sense	cgtctccagcaccctcttta
MC1R antisense	tacagaatcgccatgagtgc
MC3R sense	tctatgcccttcggtaccac
MC3R antisense	gaacatcacgccgcagat
MC4R sense	atcatgtgtaacgccgtcat
MC4R antisense	cagatgcctcccagaggata
MC5R sense	tgttcgactccatgatctgc
MC5R antisense	ggtagcgcaaggcatagaag

### MTT-Assay

Cell viability of osteoblasts was assessed on days 1, 3, 5, 7, 10, 14 and 25 adding 10 µl MTT reagent (Sigma‒Aldrich, Schnelldorf, Germany) to the medium for 2 h. Then, the formazan crystals were dissolved in 100 µl of MTT detergent (Sigma‒Aldrich) for 2 h, and the solution was measured photometrically with an ELISA reader (Sunrise, Tecan, Switzerland) at wavelengths of A550 nm and A630 nm as a reference.

### Alizarin Red S staining

On days 7, 14 and 25, the cells were fixed after washing with phosphate-buffered saline (PBS) twice and incubated for 30 min with 4% PFA at 37 °C and 5% CO_2_. PFA was removed, and the cells were washed with PBS. The mineralisation was visualized with 0.5% Alizarin Red S, pH 4 (Sigma‒Aldrich), and nonspecific stain was removed by stringent washing with PBS several times. Afterwards, the calcified matrix was dissolved in 10% cetylpyridinium chloride overnight, and the signal density was determined photometrically at a wavelength of 570 nm (A630 nm was used as a reference).

### Single-cell mRNA sequencing

Tissue preparation, sequencing and data analysis were performed as described in detail by Tikhonova et al. [42]. Briefly, for single-cell sequencing, five native femurs of 14-week-old mice or fractured femurs (d14 of healing) of female WT mice were used. Fracture surgery (closed, midshaft femur fracture stabilized with an intramedullary screw) was performed on 12-week-old mice that were sacrificed 14 days after surgery (aged 14 weeks). The surrounding soft tissue was removed carefully, as was the intramedullary nail from the fractured femurs. The metaphysis region was spatially dissected, and bones were digested (collagenase type I and IV, 2 mg/ml) for 20 min at 37°C under gentle agitation to obtain a single-cell suspension. Next, single-cell suspensions were subjected to lineage depletion using a lineage cell depletion kit (MACS, cat# 130-090-858) following the manufacturer’s instructions to enrich bone stromal cells (BSCs). BSCs were resuspended to a final concentration of 106 cells/ml in 0.05% BSA in PBS and used for droplet-based scRNA-seq using a Chromium controller (10X Genomics). The scRNA-seq libraries were prepared using a chromium single-cell 3’ regent kit (V3) (10X Genomics, cat# PN-10000075) according to the manufacturer’s protocol. The scRNAseq library was evaluated and quantified by an Agilent Bioanalyzer using a high-sensitivity DNA kit (cat# 5067 − 4626) and a Qubit (Thermo Fisher Scientific, Cat# Q32851). Individual libraries were diluted to 4 nM and pooled for sequencing. The pooled library was sequenced by using a high-output kit (150 cycles) (Illumina cat# TG-160-2002) with a Next500 sequencer (Illumina). Single-cell RNA sequencing data analysis was performed according to the procedures described in Sivaraj et al., 2022 [43]. The single-cell RNA-seq data used in this study have been deposited in the Gene Expression Omnibus (GEO) under accession numbers GSE154247 [43].

### Statistical analysis

The results are shown as the means with SDs. Individual values appear as data points in each graph. Statistical analyses were carried out using GraphPad Prism version 8.4.3 for Windows (GraphPad Software, San Diego, California, USA; www.graphpad.com). Different statistical tests were used depending on the groups that were compared and are indicated in the legend of the figures. The results were considered significant at a p-value *p* < 0.05.

## Results

### Proliferation and mineralisation of WT osteoblasts in the presence of α-MSH

**Fig. 1:**
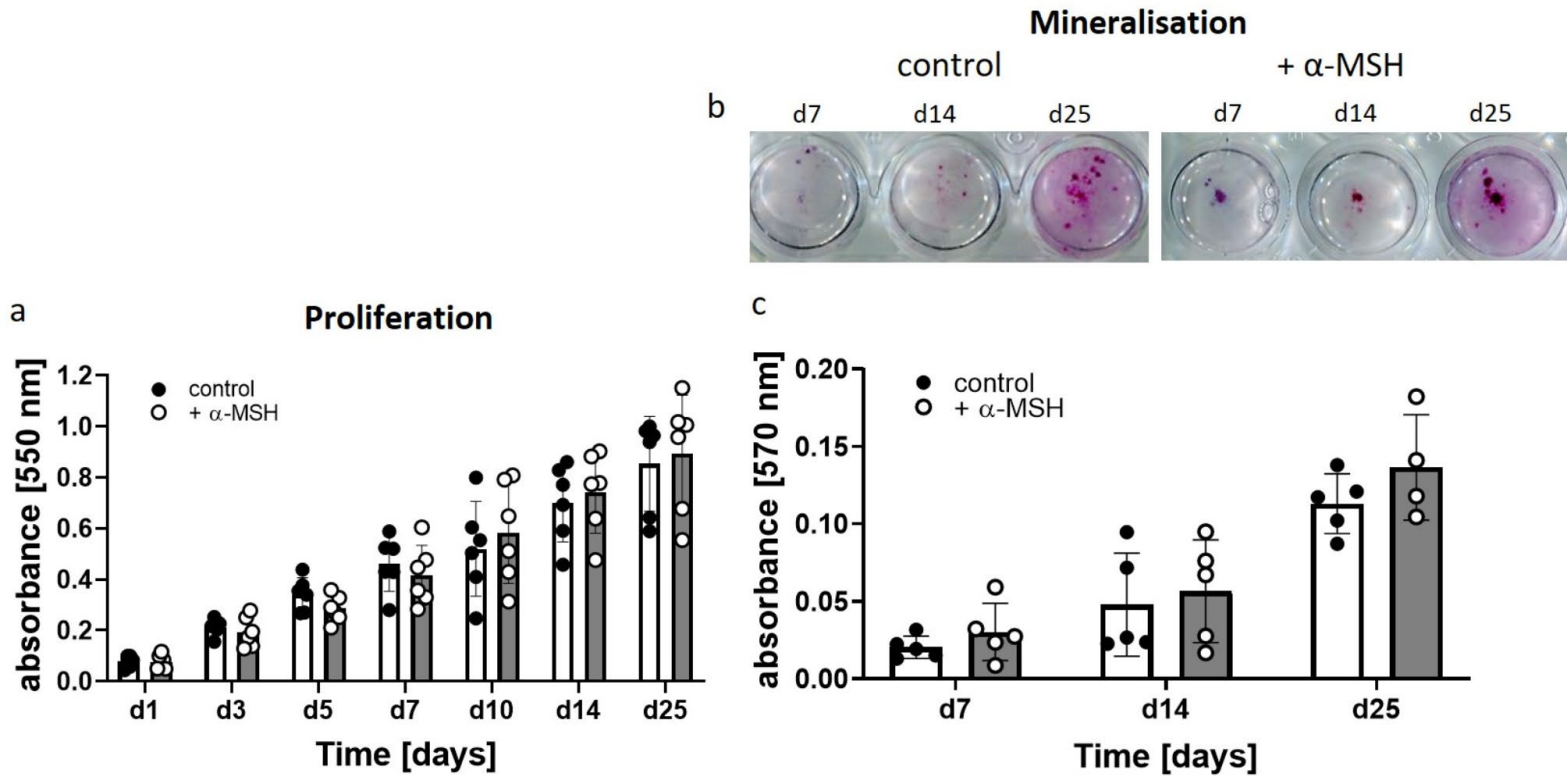
Proliferation and mineralisation (ECM) of osteoblasts is independent of α-MSH. Primary osteoblastic cells were cultured under osteoblastic differentiation conditions in the absence (control) or presence of 1 µM NDP-α-MSH (+ α-MSH). **a**: Proliferation (MTT assay) of primary osteoblastic cells during osteoblast differentiation for up to 25 days. **b**: Mineralisation of the extracellular matrix (quantified after Alizarin Red S staining) by osteoblastic cells during differentiation for up to 25 days. was quantified (*n* = 5–6 independent isolations). Statistical analysis was carried out with GraphPad Prism software using two-way ANOVA with Šídák’s multiple comparisons test. No significant differences (*p* < 0.05) were found.

Using primary murine osteoblastic cells isolated from the calvaria of newborn mice, we evaluated the effect of α-MSH on proliferation (Fig. 1a) and osteoblast function, measured as the mineralisation capacity of the extracellular matrix (Fig. 1bc). The proliferation rate of WT osteoblasts was quantified after up to 25 days of osteoblastic differentiation in the absence or presence of α-MSH. The proliferation of the α-MSH-treated cells decreased slightly at the beginning (days 3 to 7), whereas the proliferation increased slightly after day 10; however, overall, the values did not differ significantly.

The production of extracellular matrix and mineralisation during differentiation were quantified by Alizarin Red S staining, and the results are shown in Fig. 1bc. No significant differences were detected between the control group and the α-MSH-stimulated osteoblastic cell group.

### Expression pattern of melanocortin receptors in osteoblasts

**Fig. 2:**
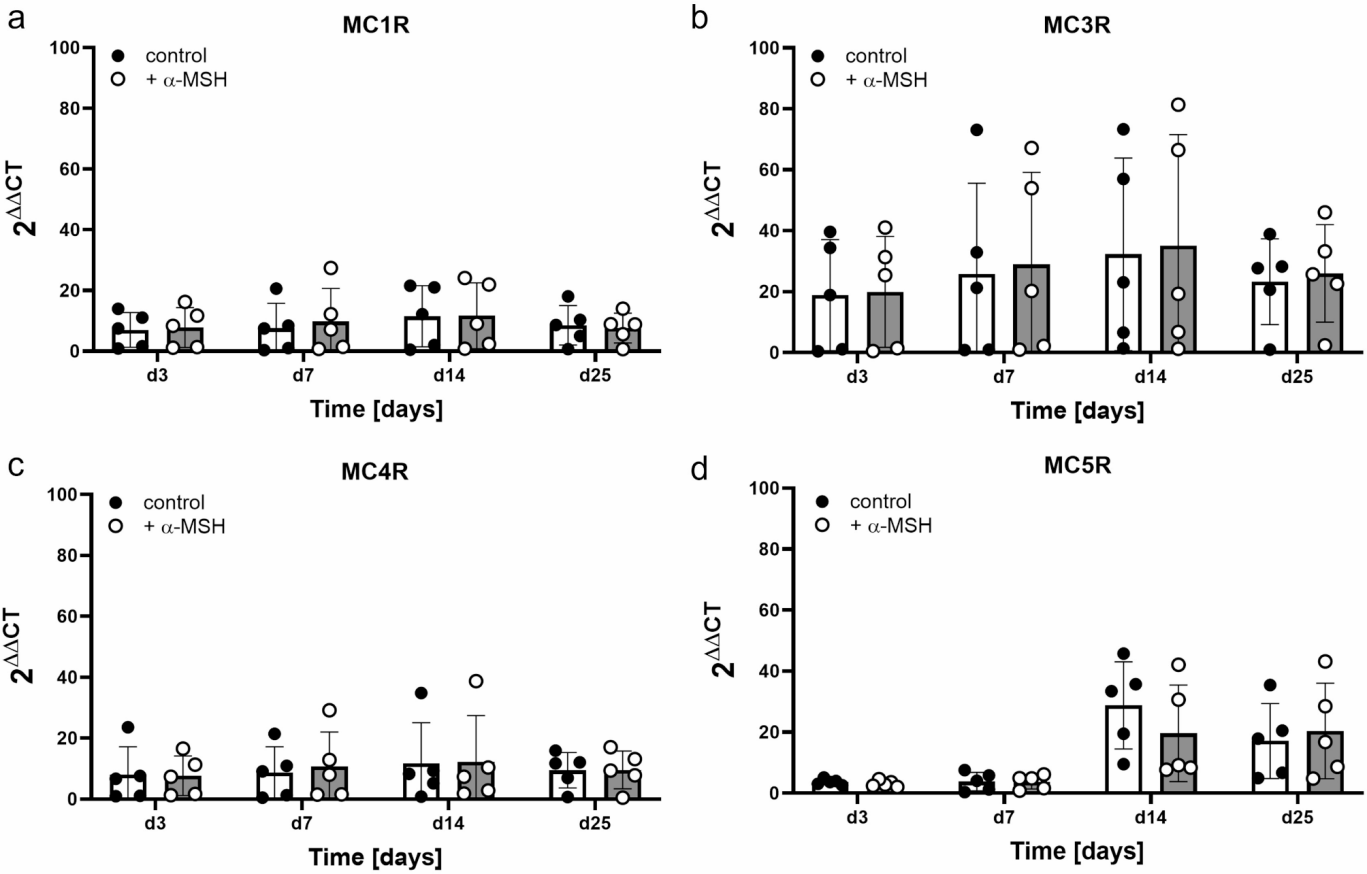
mRNA expression profile of MCR subtypes in osteoblastic cells dependent on α-MSH. Primary osteoblasts were cultured under osteoblastic differentiation conditions for up to 25 days. mRNA expression was analysed using quantitative real-time PCR, which revealed the expression of MC1R, MC3R, MC4R and MC5R in osteoblastic cells during differentiation. CT values were normalised to those of HPRT, which was used as a reference gene, and to those on day 1, which was used as the baseline. Melanocortin receptor expression was low (control) and not influenced by supplementation with 1 µM NDP-α-MSH (+α-MSH). Statistical analysis was carried out with GraphPad Prism software using two-way ANOVA with Šídák’s multiple comparisons test. No significant differences (*p* < 0.05) were found.

We studied the presence of the possible target receptors of α-MSH, melanocortin-1, -3, -4, and − 5, in WT osteoblastic cells during osteoblast differentiation at the gene expression level. As MC2R does not interact with α-MSH but only with ACTH, it was excluded from the analysis. Generally, rather low mRNA levels of each MCR (as inferred from CT values of approximately 30 to 34 cycles on day 1 as baseline, with 25 as the maximal CT value) could be detected in osteoblastic cells. Throughout the entire differentiation process (Fig. 2, white bars/black circles) of osteoblasts, a slight increase after day 14 was observed. Compared with those of the other MCRs, the expression levels of MC3R and MC5R (scale of the y-axis) were slightly elevated; MC3R mRNA expression started to increase after day 7, whereas MC5R mRNA expression increased around day 14. However, there were also experimental replicates where the expression of each receptor was near zero, as shown by single dots for replicates. Furthermore, we examined whether the expression levels could be stimulated by adding the ligand α-MSH during differentiation, but no significant differences were detected in the cells cultured in the absence of α-MSH compared to the α-MSH-enriched cell culture.

### Proliferation and mineralisation of WT osteoblast precursor cells stimulated by inflammatory cytokines in the presence of α-MSH

**Fig. 3:**
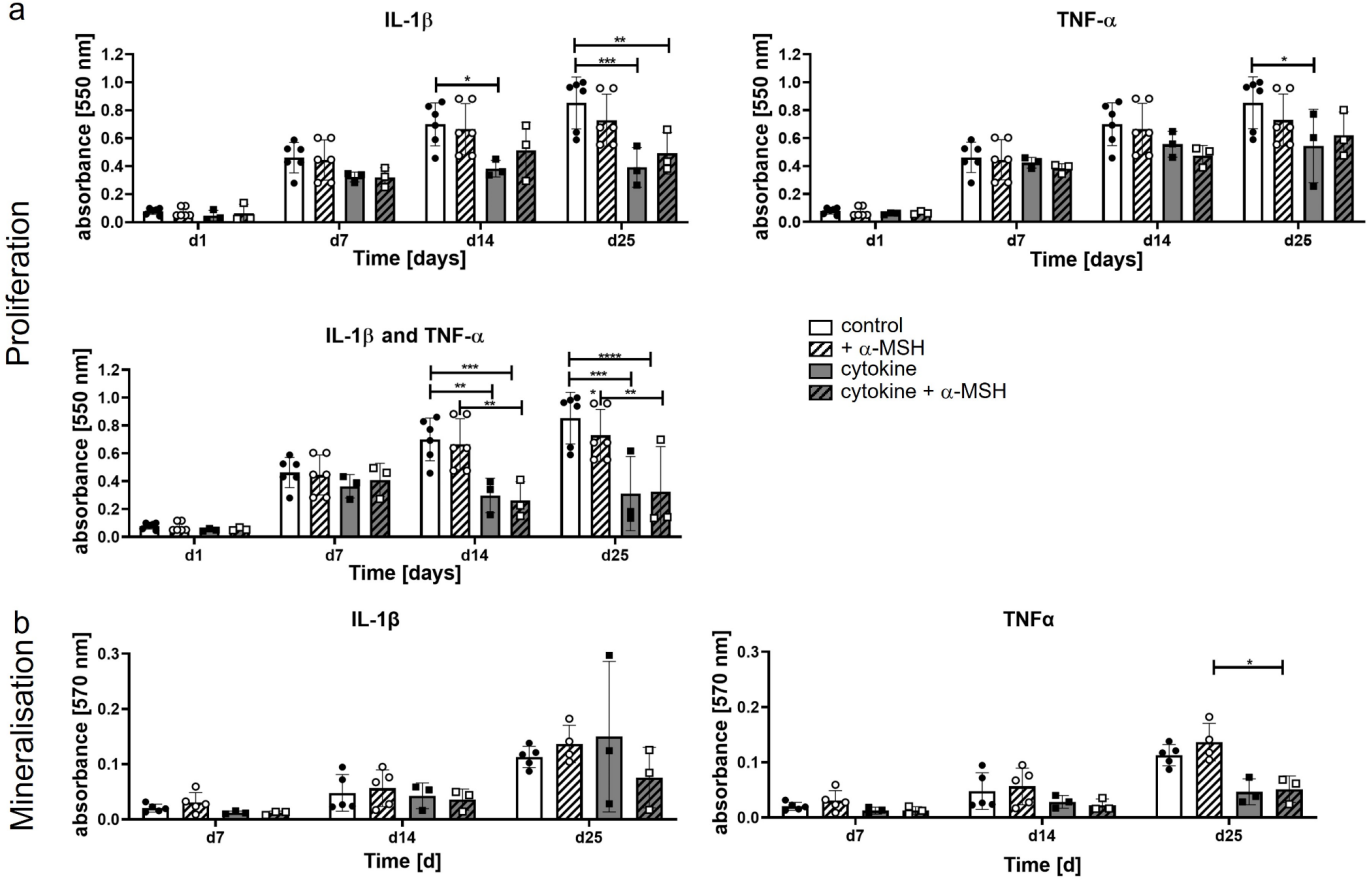
Proliferation and mineralisation (ECM) of osteoblasts (WT) are decreased by the presence of proinflammatory cytokines independent of α-MSH. **a**: Proliferation of cells determined by MTT assay is decreased in the presence of IL-1β (significantly from day 14) and TNFα (significantly on day 25). Supplementation with α-MSH (+α-MSH) had no obvious effect. The cells died in the presence of both cytokines after d7. **b**: Mineralisation of the extracellular matrix (Alizarin Red S staining and destaining measured photometrically at 570 nm) by osteoblastic cells during differentiation for up to 25 days was not influenced by Il-1β and was even decreased by TNFα. Supplementation with α-MSH (+α-MSH) had no ameliorating effect. Statistical analysis was carried out with GraphPad Prism software using 2-way ANOVA with Šídák’s multiple comparisons test (MTT) or Sidak´s multiple comparison test (Mineralisation). Significance: *p < 0.05, **p < 0.01, ***p < 0.0001, ****p < 0.00001

Stimulation with proinflammatory cytokines such as IL-1β and TNF-α in osteoblast precursor cell cultivation was used to investigate the impact of inflammation (Fig. 3, grey bars) and the influence of α-MSH (white shaded bars). The proliferation of osteoblastic cells decreased in the presence of both cytokines (10 ng/ml each), IL-1β and TNF-α, during the whole cultivation period (Fig. 3a) compared to that of unstimulated cells or cells in the presence of only α-MSH. The combination of IL-1β and TNF-α had a negative effect on cell viability. These cells died during cultivation from day 7 onward. Therefore, no further experiments were performed with a combination of both cytokines. The cytokine concentrations we used matched commonly used stimulation experiments to simulate inflammation in cell culture with different cell types. In the presence of IL-1β alone, the number of viable cells did not increase further after day 7. This effect was slightly but not significantly attenuated by α-MSH supplementation. In the presence of TNF-α, the addition of α-MSH seemed to have no effect.

Surprisingly, the mineralisation of the extracellular matrix after IL-1β stimulation did not change or even decreased (Fig. 3b, grey bars, left graph). In contrast, in the presence of α-MSH in addition to IL-1β, the mineralisation was diminished, although not significantly (grey shaded bars). The cells stimulated with TNF-α showed reduced mineralisation (grey bars, right graph), which was marginally but not significantly higher in α-MSH-treated cells (grey shaded bars) on day 25. Due to the variable growth of primary isolated cells, the high dispersion of the values should be considered.

### Influence of α-MSH in combination with cytokines on osteoblastic MCR expression

**Fig. 4:**
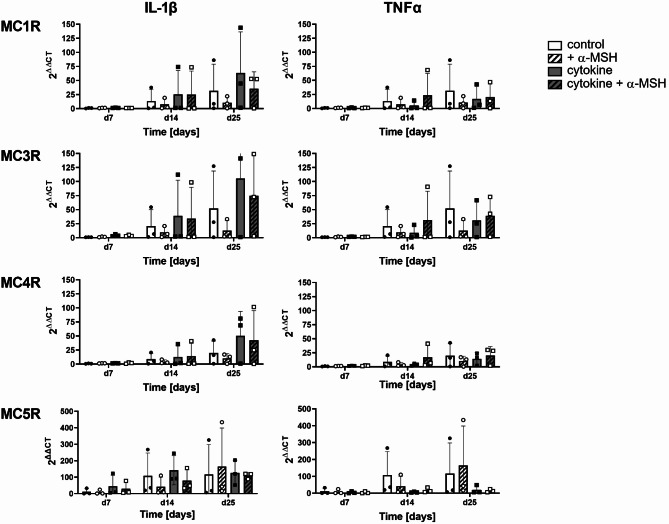
The mRNA expression profiles of MCR subtypes in osteoblastic cells are influenced by proinflammatory cytokines more than by α-MSH. The mRNA expression of MC1R, MC3R, MC4R and MC5R was analysed using quantitative real-time PCR in osteoblastic cells during differentiation for up to 25 days. CT values were normalised to those of HPRT, which was used as a reference gene, and to those on day 1, which was used as the baseline. The expression of melanocortin receptors was slightly increased (Il-1β), and its expression was decreased by supplementation with 1 µM NDP-α-MSH (+α-MSH). TNFα decreased the expression of all MCRs. Statistical analysis was carried out with GraphPad Prism software using 2-way ANOVA with Tukey´s multiple comparisons test. No significant differences (*p* < 0.05) were found.

Because of the relatively small effects observed on osteoblast viability or functionality, we examined the expression of MCR in osteoblasts stimulated with IL-1β (left) or TNF-α (right), with (shaded) or without (clear) α-MSH (Fig. 4). In the presence of IL-1β (grey bars), the expression of MC1R, MC3R, and MC4R was elevated, which was marginally attenuated by the addition of α-MSH. MC5R expression was comparable between the control (white bars) and IL-1β-stimulated cells, whereas the addition of the ligand α-MSH slightly decreased the expression of MC5R in IL-1β-stimulated cells. TNF-α-stimulated cells, independent of α-MSH treatment, showed slightly reduced expression of all receptors, which was mostly visible but also not significant in MC5R (Fig. 4). These results indicate that proinflammatory cytokines influence the mRNA expression of MCRs differently, although the mRNA expression in general was still low, and the effects were not significant.

### Effect of α-MSH treatment on bone structure in native and fractured femurs

To evaluate the systemic effect of treatment with α-MSH during bone healing on the bone structure of the unfractured right femur, microcomputed tomography (µCT) was used, and bone structure parameters were analysed. There were no significant differences in bone volume to tissue volume in general or in trabecular or cortical parameters between the femora of control mice (0.9% NaCl-treated, clear bars) and the femora of α-MSH-treated (shaded bars) mice (Supplemental Fig. 1).

**Fig. 5:**
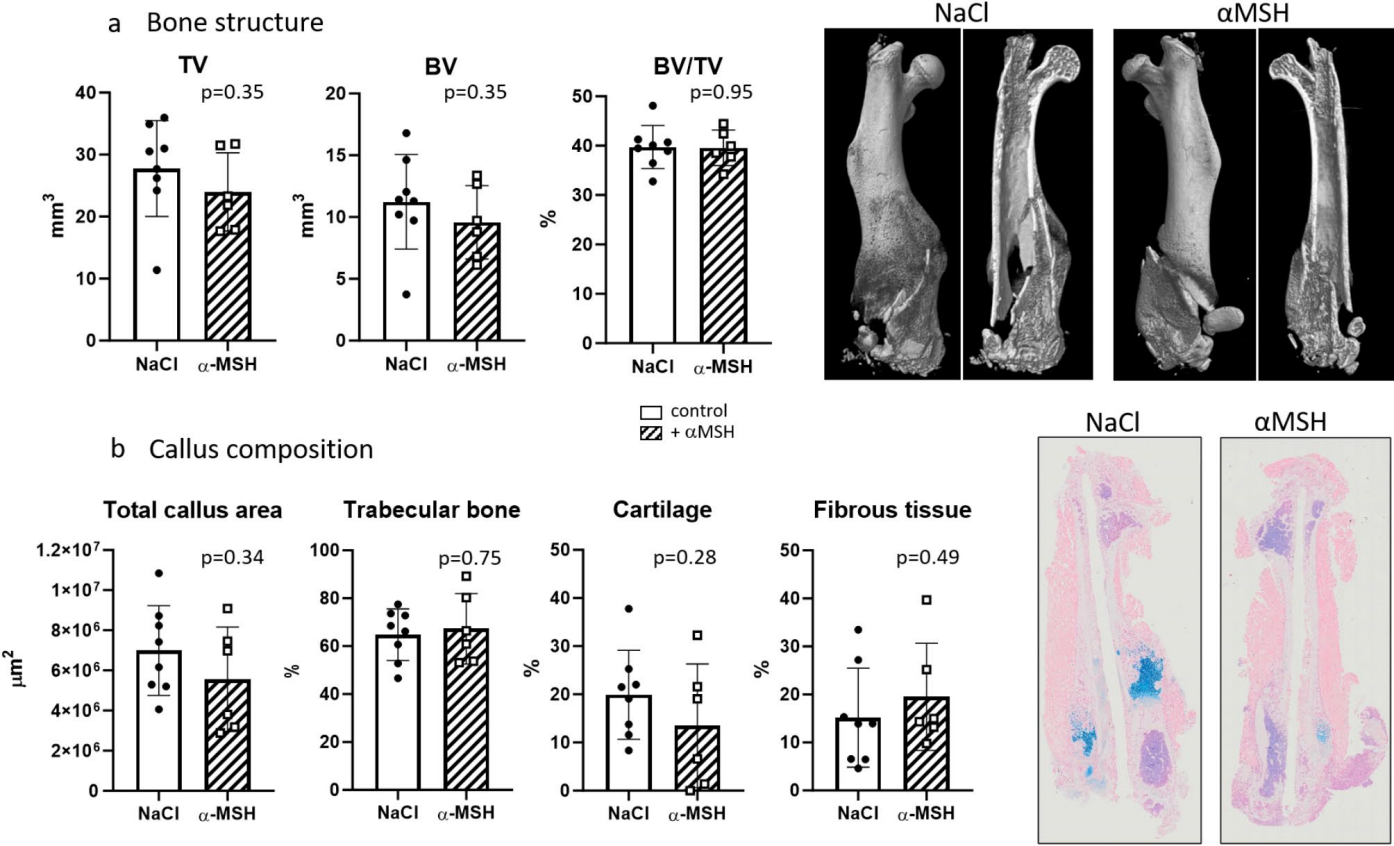
.

To evaluate the influence of α-MSH treatment on bone fracture, the bone tissue of fractured left femurs was examined (Fig. 5). Representative images of fractured bones are shown in Fig. 5 on the right side. µCT analysis of the bone structure revealed a slight but not significant decrease in the total callus size (Fig. 5a, TV, *p* = 0.35) and total bone volume (BV, *p* = 0.35) in the α-MSH-treated group compared to those in the control (NaCl) group. The BV/TV indicated that the proportion of bony callus remained similar in both untreated (NaCl) and treated (α-MSH) bones (*p* = 0.95). Histomorphometrically, as depicted in Fig. 5b (see also representative pictures of fractured bones on the right side), the callus composition, as determined by the area of the total callus, trabecular bone, cartilage, and fibrous tissue, showed a reduction in cartilage tissue, whereas the fibrous tissue fraction was slightly elevated in the α-MSH-treated callus tissue compared to that in the control group. The results for bone structure and callus tissue composition were marginal and not significantly different (p-values: Total callus area: *p* = 0.34, Trabecular bone: *p* = 0.75, Cartilaginous callus: *p* = 0.28, Soft tissue callus: *p* = 0.49).

### Single-cell mRNA sequencing analysis of the expression patterns of melanocortin receptors in native and fractured bones

**Fig. 6:**
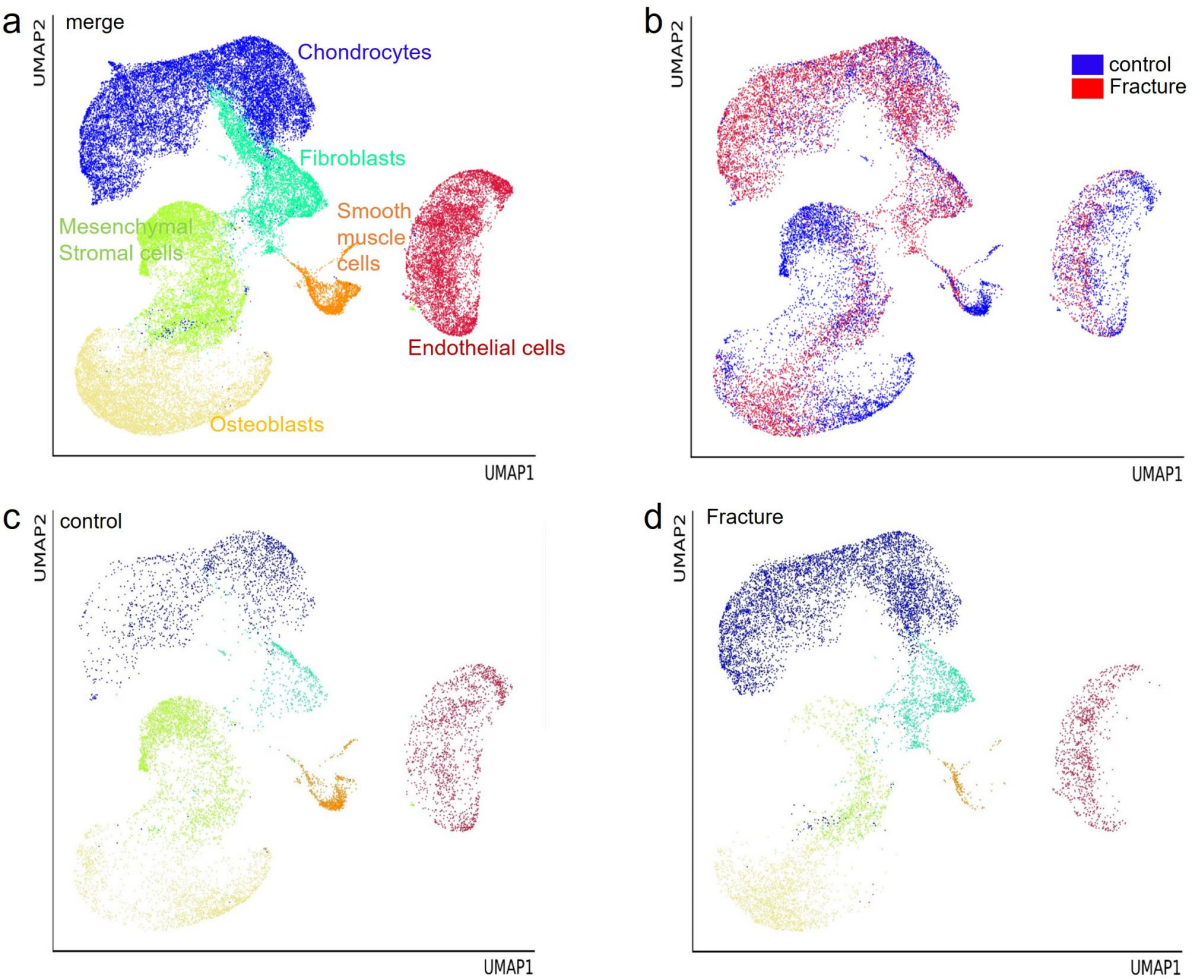
Single-cell mRNA sequencing data of the native and fractured femurs of 14-week-old mice. Tissue processing (5 mice per group) is described in the methods section. Clustering of all cells sequenced (merge) is shown in **a**. **b**: Cluster composition differentiated by the origin of cells from native bone (blue) and fractured bone (red). **c**, **d**: Cell clusters originating from cells of native bone (**c**) and fractured bone (**d**)

**Fig. 7:**
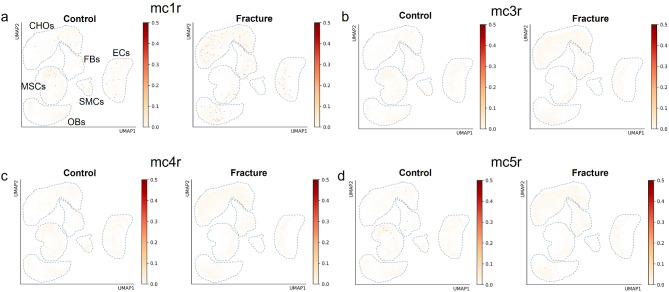
Melanocortin receptor expression pattern in cells from the tissue of native and fractured femurs of 14-week-old mice. mRNA expression of MCR in native (Control) and fractured (Fracture) bone tissue using single-cell mRNA sequencing. Clustering of cells in different cell types is shown in Fig. 6. Light yellow dots represent single cells, and dark red dots represent target gene-expressing cells. The expression of each MC receptor was low in each cell type annotated here and under both conditions analysed. The scale shows the expression level, with 0 = low and 3 = max.

We took advantage of access to a dataset from single-cell mRNA sequencing of native and fractured bones after 14 days of healing [42, 43] and examined the mRNA expression of MCR molecules at single-cell resolution. In Fig. 6a-d, the clustering of different cell types is shown as a merged image of all cells (a) and as an overview of cells in native (b, blue dots) and fractured bones (b, red dots). In Fig. 6c-d, the cell amount in each cell cluster is shown dependent on the conditions of native (c) and fractured (d) bones. As shown in Fig. 7a-d, the mRNA expression patterns of MC1R (a), MC3R (b), MC4R (c), and MC5R (d), depicted as yellow to red dots clustered in various cell types of the bone, were analysed. However, the number of cells expressing one of the different MCRs was very low in native bones as well as in bones after fracture. After fracture, we detected an increase in the number of MC1R-expressing cells among several cell types, such as chondrocytes, osteoblasts, and endothelial cells, but the number of cells was still very low. MC3R, MC4R, and MC5R expression was very low (MC5R) or could not be detected within the cells of native or fractured bones. This could be due to very low expression levels of these proteins and fits our results using cells from in vitro culture.

## Discussion

α-MSH is known to be involved in various processes in the body, such as inhibiting chronic inflammation [5–10]. In the musculoskeletal context of rheumatoid arthritis and osteoarthritis, the anti-inflammatory effects of α-MSH on synovial fibroblasts and chondrocytes have been well described, but only a few studies have been carried out regarding the impact of the melanocortin system on osteoblasts and bone or bone regeneration [15–17, 31]. RA patients, as well as patients suffering from other systemic chronic inflammatory diseases or aged humans, have an increased risk of impaired fracture healing that is not well understood to date, but elevated systemic levels of inflammatory cytokines may play a significant role in this process [44, 45]. As bone healing initiates with an essential inflammatory phase, anti-inflammatory treatment should not interfere with these processes of regeneration. To evaluate potential side effects of α-MSH treatment on bone, we aimed to understand the impact of α-MSH and the melanocortin system on bone and investigated the influence of α-MSH on osteoblast function and bone regeneration with special attention given to the presence of proinflammatory cytokines and inflammatory processes.

We performed osteoblast differentiation experiments in the absence and presence of α-MSH and we did not find any significant difference or impairment in terms of proliferation or ECM mineralisation. Previous studies by other groups reported a dose-dependent increase in proliferation and cell number in rat osteoblasts and enhanced proliferation and mineralisation in the osteoblast-like cell line MC3T3-E1 when cultured with α-MSH [17, 46]. Although we used standard osteoblast differentiation methods, we cannot exclude the possibility that these differences might be due to the utilized cell type (primary cells vs. cell line), different doses of α-MSH, different time points or different cultivation methods. These appeared to be highly variable in this field and therefore difficult to compare. We can conclude from our results that α-MSH has no negative effect on the proliferation of primary murine osteoblasts, which is meaningful for the treatment of inflammatory diseases caused by this component.

To simulate inflammatory stress, we stimulated osteoblasts with the proinflammatory cytokines IL-1β and TNF-α in the absence or presence of α-MSH. In the presence of both cytokines, the proliferation of primary osteoblasts was inhibited or even prevented, which was expected, at least to some extent, from the literature [47–50]. The presence of α-MSH did not rescue this effect in any way. Osteoblast function in terms of mineralisation of the ECM was not reduced by IL-1β alone but was reduced in the presence of both IL-1β and α-MSH, whereas TNF-α disturbed mineralisation independently of the hormone. Similar but not equal effects of proinflammatory cytokines might be caused by shared pathways (Runx2/osterix pathway), which lead to different impacts on gene expression (IL-1β reduces the number of osteoblasts able to proliferate; TNF-α reduces osteonectin and osteopontin gene expression) and differentiation in osteoblasts [51]. In summary, regarding the impact of αMSH on osteoblasts, we confirmed a negative influence of IL-1β and TNFα, and the addition of α-MSH did not ameliorate the impact of inflammation on osteoblasts but did not have a negative impact either.

As there is little detailed information about the presence of MCRs in osteoblasts, we addressed the question of which MCR subtype might be most prominent in osteoblasts. The MCR expression pattern during osteoblast differentiation was analysed. In contrast to human (MC1R, MC4R [15]), and rat (MC4R, MC5R [52]), primary osteoblasts, we detected all known α-MSH-affinitive melanocortin-receptors in primary mouse osteoblasts, from which MC3R and MC5R were the most dominant, but all were at rather low levels. Stimulation with α-MSH as a ligand did not affect the mRNA expression levels of any of the receptors, indicating that there is no regulatory impact of α-MSH on receptor expression in osteoblasts in mice. In the presence of IL-1β, we observed elevated mRNA expression of all MCRs except for MC5R, while TNF-α had a reducing effect. This effect was described similarly by Funasaka et al. in human melanocytes [53]. The increase in receptor expression correlates with cytokine modulation in mineralisation: IL-1β slightly increases, TNF-α slightly decreases mineralisation or expression and the ligand attenuates these effects. These results were limited by high dispersion due to the variable growth of primary cells. Further research might be needed to elucidate the underlying regulatory mechanism by which IL-1β influences MCR expression and why IL-1β in combination with α-MSH has negative effects on mineralisation capacity of osteoblasts. In summary, from our in vitro experiments with isolated primary murine osteoblastic cells, we concluded that α-MSH has no measurable effect on proliferation or differentiation under physiological or inflammatory conditions and therefore did not impair osteoblast function. However, this may be different in other cell types or more complex systems with interacting cells. As chondrocytes are an important cells type in bone development or regeneration, we also investigated the chondrocyte expression of MCR and found at least increased basal levels of all MCRs and some upregulation of MC3R and MC4R expression in the presence of IL1β and α-MSH (Supplementary Fig. 4). The impact of α-MSH and MCR on chondrocytes was already investigated in the course of RA and OA in vivo and in vitro [13, 14, 27–30] and is not in the focus of this study.

Our in vitro observations were transferred and verified by examination of callus formation during fracture healing where cartilage and bone are the most important tissue components. We investigated the impact of α-MSH on callus formation during fracture healing in an in vivo mouse model. No obvious differences were found in the composition of the callus under treatment with α-MSH. The bony callus remained similar. However, a slight shift from less cartilaginous callus toward a greater fibrous tissue fraction was detected in α-MSH-treated mice.

Taken together, these findings indicate that the application of α-MSH during fracture healing leads to a slight delay in the transition of fibrous tissue callus to cartilaginous callus but does not alter the progression of trabecular callus formation. Lange et al.. described the subtle effects of additional IL-1β injections on fracture healing [54], but our results revealed no stimulatory or even negative effects of the application of α-MSH during fracture healing. We concluded that the melanocortin system might have further regulatory effects on cell types other than osteoblasts (e.g., chondrocytes, fibroblasts, and mesenchymal stromal cells), which might be worth studying in detail. Moreover, systemic administration of α-MSH for 14 days did not suggest any changes in bone volume or bone structure in the contralateral, native femora of our treated animals. This finding is consistent with the rodent study (rat) of Lim et al., who reported no bone gain or loss in male rat femora after 4 weeks of injecting α-MSH analogs [16]. On the other hand, in male Swiss mouse tibiae, Cornish et al.. reported a 22% decrease in trabecular bone volume with a decrease in trabecular number, increase in trabecular separation and overall increase in bone turnover after subcutaneous injections for 20 days [46]. Both studies differ in the strain/species used (Sprague–Dawley rats, Swiss mice, C57BL/6 mice), sex (male vs. female) and methods (2D histomorphometry vs. histomorphometry and 3D µCT) which limited the comparability of the results.

Our study had several limitations. It is possible that the high dosage of cytokines used in our in vitro experiments, referred to as high-grade inflammation, led to a functional impact on α-MSH. On the other hand, the dose of α-MSH in vivo remains unknown, so a variety of treatments could be useful. The dose of α-MSH we used in vivo was shown to be effective [39] but not in the context of bone regeneration. Using α-MSH in a RA model combined with fracture healing could be interesting, to determine whether α-MSH ameliorates the course of RA and positively influences fracture healing, which has been shown to be impaired by RA [34]. The fracture model we chose (closed midshaft fracture stabilized with an intramedullary screw) is known to result in less standardized fracture sites (fragmented fractures, location of fracture near distal ends, variation in callus size) compared to modes using osteotomy stabilized with an external fixator. This might be a disadvantage when quantifying callus tissue. In contrast, osteotomy is performed by open surgery with disturbances in the formation of the fracture hematoma by flushing and callus size is limited when a small fracture gap is applied. It therefore seemed to be more important to use a model that ensures an undisturbed initial inflammatory phase, as we started our treatment on the day of surgery, and sufficient callus size to evaluate callus composition. Additionally, the chosen fracture model represents the most similar fracture to human fracture seen in the hospital. Furthermore, the effect of sex on the melanocortin system has not been investigated thus far. In contrast to other studies that used male mice only, we performed our fracture experiments exclusively with female mice. Sex influences bone turnover, and the coincidence of chronic inflammation and osteoporosis might also play a role in the degree of impact or outcome effect related to the melanocortin system. Methodically, using µCT data enables the generation of 3D images instead of 2D images used in histomorphometry in comparative studies, leading to differences in the results. Moreover, the high dispersion of results, such as what we often observe when using primary cells, must be taken into account and limits comparability.

## Conclusion

Recent research suggests that treatment with α-MSH, as an anti-inflammatory modulator, could be a promising approach for rheumatoid arthritis treatment. Given that RA patients may also experience bone loss and impaired fracture healing, this study aimed to investigate the impact of α-MSH and its receptors in murine bone, particularly in primary osteoblasts, under physiological and inflammatory conditions, as well as in the context of bone regeneration. Our findings indicate no adverse effects of α-MSH on cellular function in osteoblasts in vitro or on bone or fracture healing in vivo, with or without inflammation. Taken together, when assessing the effect of α-MSH treatment on bone in inflammatory diseases, our study suggested that there was no impairment of bone metabolism or fracture healing.

## Electronic supplementary material

Below is the link to the electronic supplementary material.

Supplementary Material 1

## Data Availability

The single-cell RNA-seq data used in this study have been deposited in the Gene Expression Omnibus (GEO) under accession number GSE154247.
