# Supplementary material for: Anti-inflammatory treatment using alpha melanocyte stimulating hormone (α-MSH) does not alter osteoblasts differentiation and fracture healing
Source: BMC Musculoskelet Disord. 2025 Feb 6;26:123. doi: 10.1186/s12891-025-08374-9 (PMC11800508; doi:10.1186/s12891-025-08374-9)
Supplement: Supplementary file 1 — Supplementary Material 1 [file 12891_2025_8374_MOESM1_ESM.pdf]

Alpha melanocyte stimulating hormone (α-MSH) treatment does not alter bone metabolism and fracture healing

Johanna Graue[1], Melanie Timmen[1], Katharina Schmitz[1], Daniel Kronenberg[1], Markus Böhm[2], Kishor K. Siveraj[3], M. Gabriele Bixel[3] and Richard Stange*[1]

[1]Department of Regenerative Musculoskeletal Medicine, Institute of Musculoskeletal Medicine, University of Muenster, Albert-Schweitzer-Campus 1, D3, 48149 Muenster, Germany

[2]Department of Dermatology, University Hospital Muenster, Von-Esmarch-Str. 58, 48149 Muenster, Germany

[3]Max Planck Institute for Molecular Biomedicine, Röntgenstraße 20, 48149 Muenster, Muenster, Germany

Corresponding author: Richard.Stange@ukmuenster.de

Supplemental Material

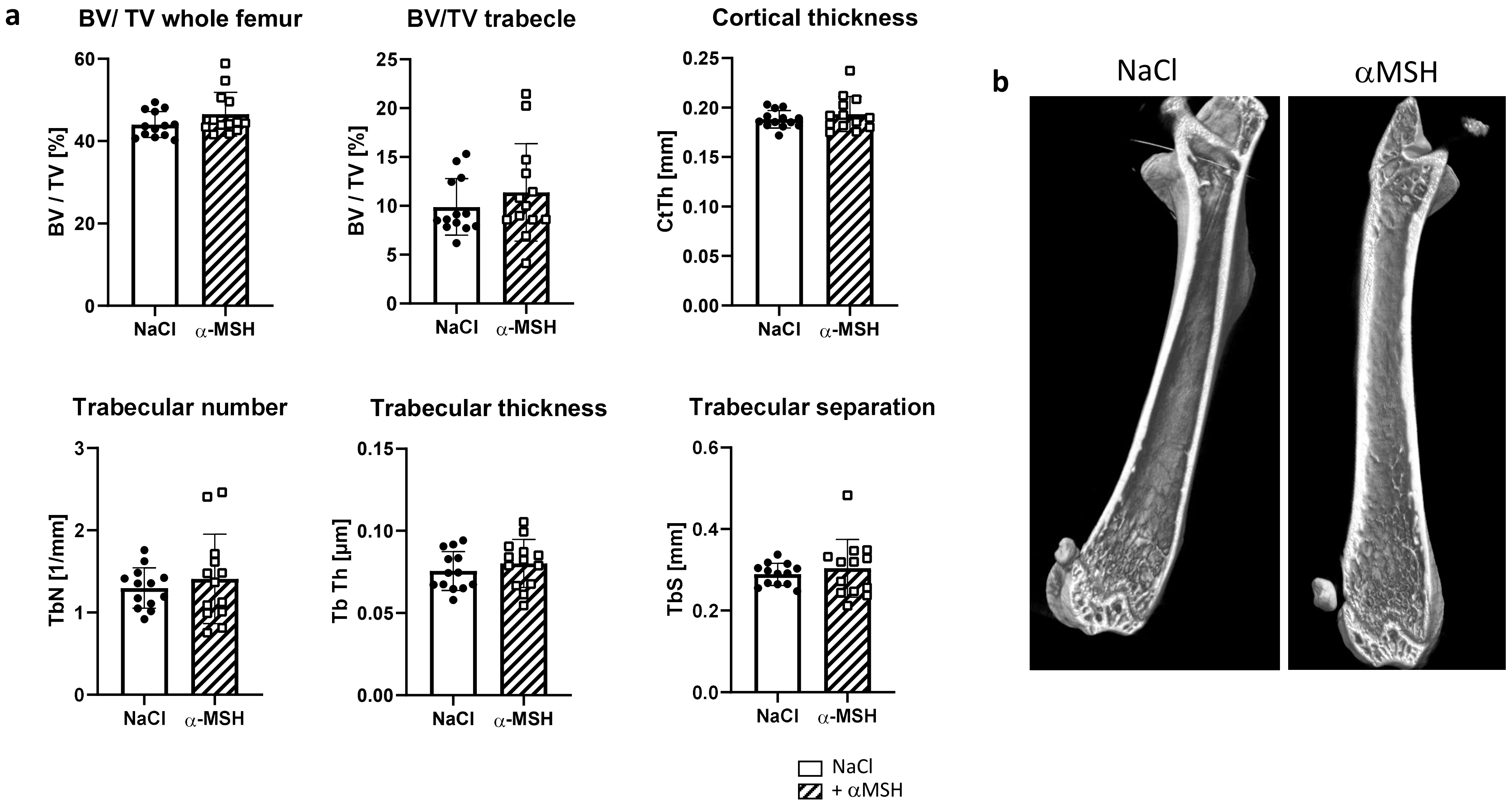

Supplemental Fig. 1 Effect of α-MSH administration on bone structure in mice. Mice underwent a closed midshaft femur fracture stabilized with an intramedullary screw and healed for 14 days. As treatment mice were injected daily (i.p.) with either 100 µl 0.9% NaCl (n=8) or 0.01mg/ml NDP-α-MSH (n=6). After sacrifice, bone structure of both femurs was analysed using µCT. a: Quantification of bone structure. b: Representative µCT images of right unfractured femurs after treatment with NaCl or α-MSH.

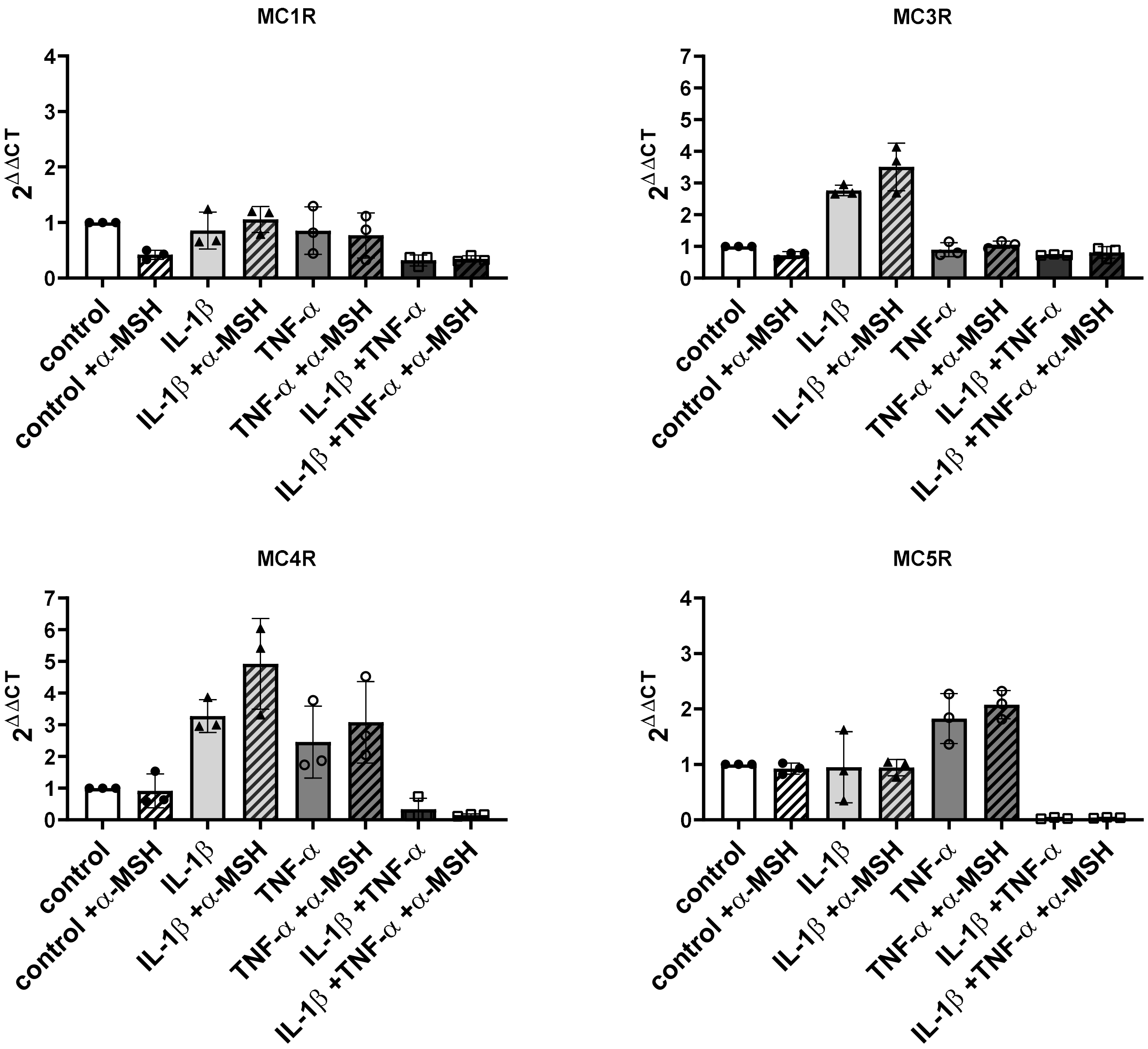

Chondrocytes

Supplemental Fig. 2 mRNA expression profile of Melanocortin receptor molecules in chondrocytes dependent on α-MSH as well as proinflammatory cytokines. Chondrocytes were isolated from sternum of 3-5 days old mice, after Trypsin/collagenase IV digestion and cultured in DMEM medium for one week. After passage, cells were allowed to adhere for 24h and than stimulated for 48h in presence of αMSH, IL-1β and/or TNFα. mRNA expression was analyzed using quantitative real time PCR revealed the expression of MC1R, MC3R, MC4R and MC5R.
